# Native Valve Infective Endocarditis with Osteomyelitis and Brain Abscess Caused by *Granulicatella adiacens* with Literature Review

**DOI:** 10.1155/2019/4962392

**Published:** 2019-07-30

**Authors:** Sachin M. Patil, Niraj Arora, Peter Nilsson, S. J. Yasar, Dima Dandachi, W. L. Salzer

**Affiliations:** ^1^Infectious Disease Department, University of Missouri Hospital and Clinic, 1 Hospital Dr, Columbia, MO 65212, USA; ^2^Neurology Department, University of Missouri Hospital and Clinic, 1 Hospital Dr, Columbia, MO 65212, USA; ^3^Internal Medicine Department, University of Missouri Hospital and Clinic, 1 Hospital Dr, Columbia, MO 65212, USA; ^4^Cardiology Department, University of Missouri Hospital and Clinic, 1 Hospital Dr, Columbia, MO 65212, USA

## Abstract

*Granulicatella adiacens* is a type of NVS (nutritionally variant streptococci) rarely causing infective endocarditis (IE). NVS are fastidious and unable to sustain growth on routine culture media due to lack of specific nutrients. Endocarditis caused by NVS due to their virulence is associated with higher treatment failures and mortality rates. New antimicrobial susceptibility patterns are indicative of a significant rise in penicillin resistance and susceptibility differences between NVS subspecies. Initial empirical therapy is essential as a delay in using the appropriate agent leads to poor results. We present a case of an immunocompetent young female with recent intravenous drug abuse resulting in native mitral valve endocarditis with ruptured chordae tendineae and septic embolization, causing brain abscess and lumbar spine osteomyelitis. She was transferred to a tertiary center where she underwent mitral valve replacement successfully and treated with six weeks of intravenous vancomycin and ertapenem. To our knowledge, ours is the first case report of *G. adiacens* endocarditis in an adult with brain abscess and osteomyelitis with an excellent response to antibiotic therapy. Based on our case report, literature review, and new antimicrobial susceptibility patterns, updates to treatment guidelines are suggested to improve the therapeutic outcomes.

## 1. Introduction

Infective endocarditis is a severe infection affecting the endocardium and heart valves resulting in significant mortality and morbidity. The etiologic agent causing infective endocarditis is an important prognostic marker. As per the latest clinical data, streptococci are responsible for 30% of cases [1]. Even with improved diagnostic and curative approaches, mortality remains high (17%) if the causative agent is less common as in our case with NVS (nutritionally variant streptococci) *Granulicatella adiacens*. NVS are responsible for 5% of cases overall caused by streptococci [2]. NVS initially detected in 1961 [3] were divided into genera *Abiotrophia* and *Granulicatella* based on 16S rRNA gene sequencing in 2000. Three species of *Granulicatella* described are *G. adiacens*, *G. elegans*, and *G. balaenopterae* [4]. On review of the medical literature, we found only a few cases of infective endocarditis caused by *Granulicatella adiacens* [5]. *Granulicatella adiacens* implicated in a single instance of brain abscess in a child with congenital heart defects with no cardiac vegetations as seen on imaging [6]. Here, we report the first case in an adult wherein native valve infective endocarditis caused by *Granulicatella adiacens* was associated with brain abscess and osteomyelitis with no prior history of brain surgery.

### 1.1. Case Report

A 44-year-old female patient admitted to the university hospital for new-onset left-sided weakness and right-sided headache; dizziness, confusion, chest pain, and palpitations for four days; and generalized malaise for two months. Medical history was significant for hypertension, chronic hepatitis C treatment naive, hypothyroidism, recent IV drug abuse, and alcoholism, and she was a former heavy smoker. A CT scan (computerized tomography) of the head done at the outside hospital revealed acute infarctions in the right thalamus and right temporal lobe. EKG showed normal sinus rhythm, and a chest X-ray was normal.

**Figure 1:**
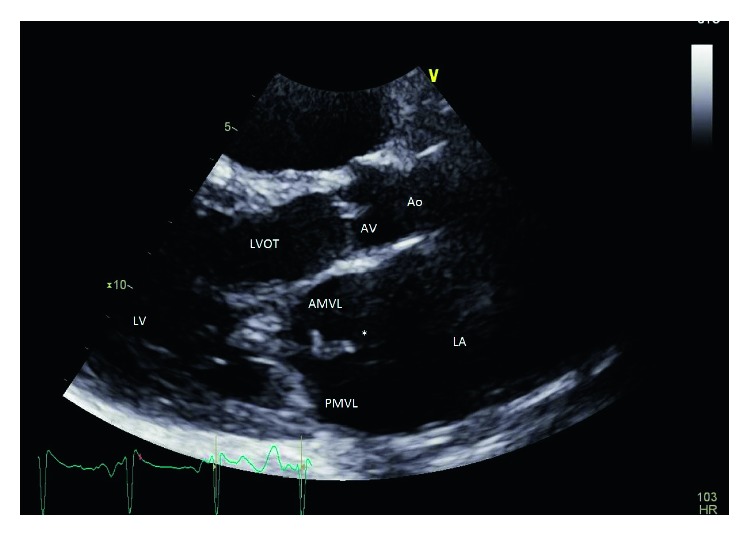
Transthoracic echocardiogram in parasternal long-axis view. Vegetation emanating from the anterior mitral valve leaflet is marked by an asterisk. LA: left atrium; LV: left ventricle; LVOT: left ventricular outflow tract; AV: aortic valve; Ao: aorta; AMVL: anterior mitral valve leaflet; PMVL: posterior mitral valve leaflet.

**Figure 2:**
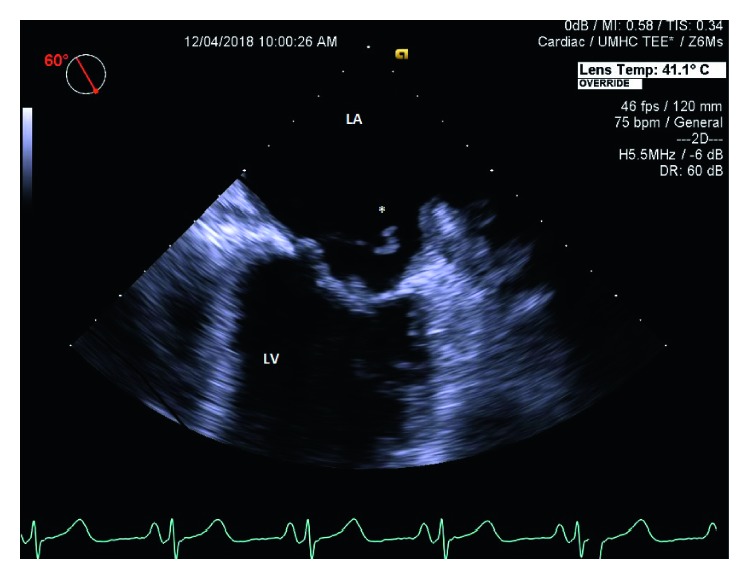
Transesophageal echocardiogram at the mid-esophageal level with 60 degree rotation. Ruptured chordae tendineae from the posterior mitral valve leaflet (marked with an asterisk).

On the day of admission, vital signs were blood pressure of 118/74 mmHg, pulse rate of 117/minute, respiratory rate of 20/minute, temperature of 38°C, and SpO_2_ of 98% on room air. Physical examination revealed the presence of poor oral hygiene and grade 3/6 systolic murmur at the cardiac apex. Neurological examination affirmed slurred speech, left-sided weakness with motor strength of 1/5 in upper and lower extremities, increased reflexes on the left side compared to the right, and right pupil dilation with sluggish response to light and right lateral ophthalmoparesis. EKG showed sinus tachycardia. TTE (transthoracic echocardiogram) (Figure 1) displayed an ejection fraction of 65%, dilated left atria, and severe mitral valve regurgitation with a 2 cm strand-like hypoechoic structure on its atrial surface suggestive of vegetation. CT angiogram of the head and neck displayed the patent carotid and vertebral basilar arterial system. Multifocal acute infarcts were detected at the right medial temporal lobe, right thalamus, right lateral pons, and midbrain with no hemorrhagic transformation on MRI (magnetic resonance imaging). TEE (transesophageal echocardiogram) (Figure 2) on day 4 revealed no evidence of thrombus or mass, ruptured chordae tendineae of the posterior mitral leaflet, and a small mobile density on the anterior mitral leaflet with no patent foramen ovale. Blood cultures obtained on day 3 of hospitalization for fever of 38.7°C resulted positive on day 5 for Gram-positive cocci in chains in 4 out of 4 bottles.

The infectious disease team was consulted on day 4 of admission. The intravenous (IV) antibiotic vancomycin 1.5 g every 12 hrs was initiated on day 5 to achieve a target vancomycin trough of 15 to 20 mcg/ml. Overall, blood cultures obtained on days 3 and 5 resulted positive in 4 out of 4 bottles. MALDI-TOF MS (matrix-assisted laser desorption ionization-time of flight mass spectrometry) was used to identify the organism due to difficulty in retrieving it from culture media. *G. adiacens* was confirmed on day 6 in all of the positive blood culture samples obtained. Isolated colonies determined insufficient to be transferred to a tertiary lab for antimicrobial susceptibility. IV vancomycin was continued as per treatment plan. From day 6 onwards, blood cultures remained negative for any growth. A cardiothoracic surgeon evaluated the patient for mitral valve replacement/repair surgery.

**Figure 3:**
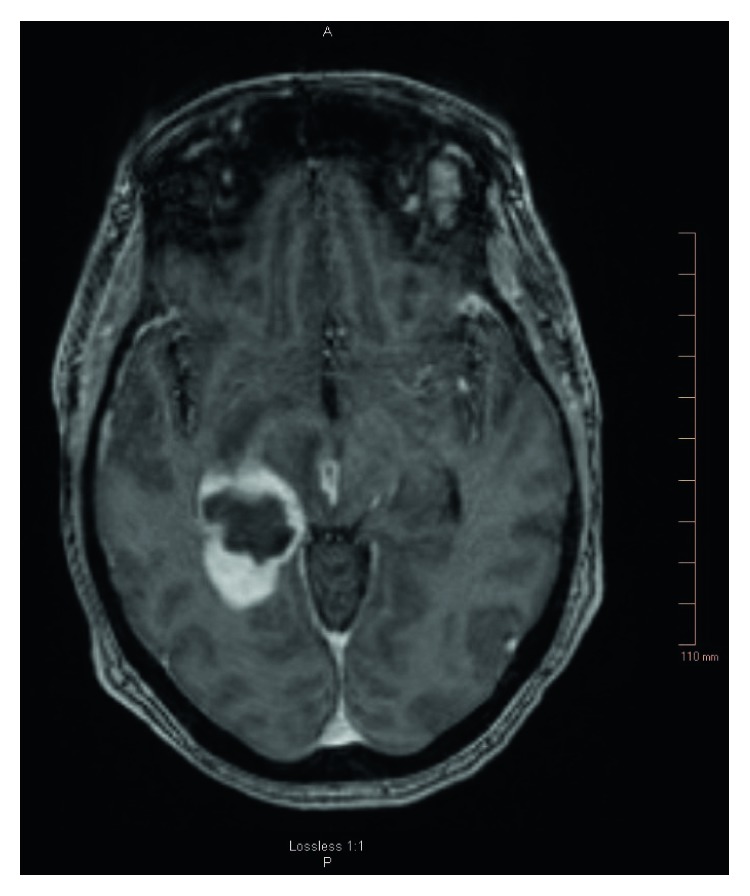
MRI brain T1 MPR TA reveals right thalamic and right medial-temporal/occipital parenchymal abscesses with extensive vasogenic edema.

**Figure 4:**
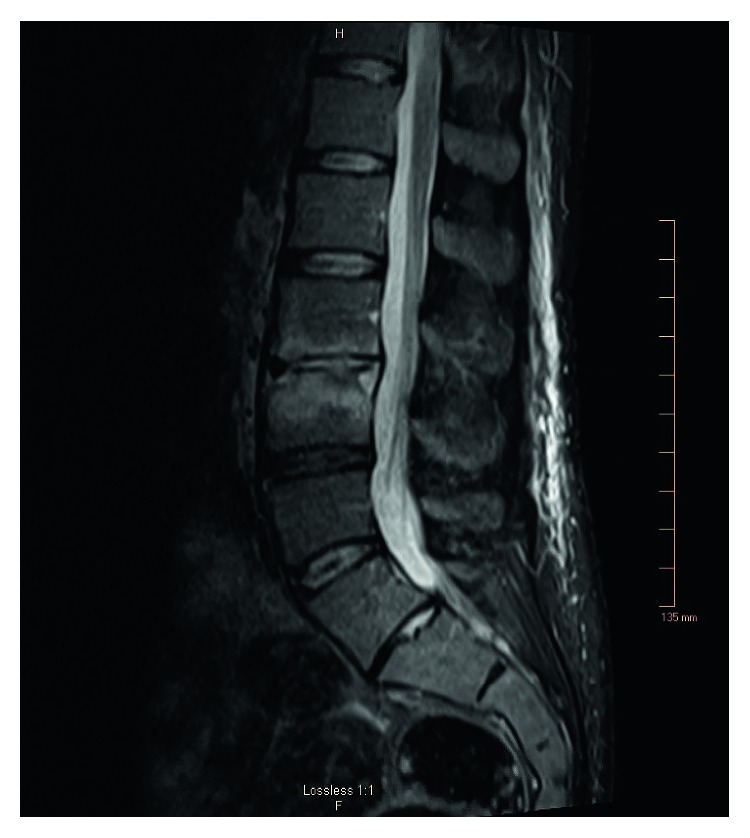
MRI lumbar spine reveals L3-L4 discitis and osteomyelitis.

On day 9, the patient complained of acute low back pain. A CT scan of the lumbar spine with contrast revealed L3-L4 early discitis and osteomyelitis. On the same day, the patient's confusion worsened and was transferred to the neuroscience intensive care unit. The MRI brain was repeated for deteriorating confusion. It (Figure 3) revealed right thalamic and right medial temporal-occipital abscess (measuring 1.1 × 2.3 × 2.9 cm) associated with extensive vasogenic edema extending to the brainstem. Lumbar spine MRI (Figure 4) displayed L3-L4 discitis with osteomyelitis with no epidural or paravertebral abscess. The CSF (cerebrospinal fluid) analysis revealed an elevated total protein of 196 mg/dl, decreased glucose of 33 mg/dl, elevated white cell count of 385/mcl, lymphocyte predominant (85%), and RBC of 1040/mcl. The CSF BioFire panel for meningitis pathogens was negative. At this time, IV meropenem 2 g every 8 hrs was started alongside with vancomycin. The patient's confusion gradually cleared with improvement in speech and left-side strength. Cardiac catheterization revealed normal coronary arteries without any significant disease. Normal coronary arteries implied that ruptured chordae tendineae was due to infection. Cardiothoracic surgery reevaluated the patient and recommended transfer to a larger tertiary care facility due to the complex clinical condition and high surgical risk. Vancomycin trough (mcg/ml) was 14.6 on day 7, 21.4 on day 14, and 24.8 on day 20.

The tertiary care center transfer was on day 21 in a clinically stable condition. IV vancomycin and meropenem continued at transfer. Mitral valve replacement with Hancock type 2 MV tissue was done on day 31, and the patient was discharged on six-week course of IV vancomycin and ertapenem. On clinical follow-up at three months, mild residual weakness on the left side was noted, and brain imaging revealed resolving abscesses.

## 2. Discussion

NVS detected as small satellite colonies near larger colonies of helper bacteria such as *Staphylococcus* and *Hemophilus influenzae* were implicated as causative agents of endocarditis and otitis media in 1961 [7]. Unable to synthesize essential nutrients such as pyridoxal and L-cysteine, they exhibit microbial commensalism [2]. NVS were classified initially as a separate genus *Abiotrophia* in the mid-1990s [8], and this genus has been divided into the families *Abiotrophia* and *Granulicatella* based on 16S rRNA gene sequencing. *Granulicatella* are catalase negative and oxidase negative facultative anaerobic Gram-positive cocci. They are seen as Gram-positive cocci or cocobacilli in chains in optimal nutritional conditions and pleomorphic with a globular and filamentous form in poor nutritional conditions [7].

NVS are a part of the healthy oral flora, urogenital system, and intestinal tract [9, 10]. *G. adiacens* are observed more frequently in the oral cavity and are found in dental plaques, endodontic infections, and dental abscesses [11–15]. Virulence is attributed to specific characteristics of NVS. *A. defectiva* and *G. adiacens* carry Cha gene responsible for producing Cha protein which binds to fibronectin [16]. Cha protein has fibronectin-binding activity in the repetitive and unique area with a higher affinity of the unique region. *A. defectiva* strains have a higher affinity to bind to ECM (extracellular matrix) rich in laminin [17]. The decreased binding ability of *G. adiacens* to ECM components explains lower rates of infective endocarditis compared to *A. defectiva* [18]. Endovascular infectivity of *G. adiacens* is related to its fibronectin-binding capacity, an essential process for bacterial adherence, initiation, and sustaining endovascular bacterial adhesion in infective endocarditis and dissemination of infection [16, 17]. Due to nutrition limitation in cardiac vegetations, NVS grow slowly leading to structural abnormalities such as thick cell walls, filament formation, and increased exopolysaccharide production [19]. This leads to treatment difficulty necessitating a prolonged course of antimicrobial therapy for complete cure. NVS exhibit PCN (penicillin) tolerance [7].

Infective endocarditis due to NVS is subacute in onset, classic endocarditis signs are seen rarely, and vegetations are smaller with prominent embolization [20]. In a case series of *G. adiacens* related endocarditis, the aortic valve was most commonly involved in 44% of the cases, followed by the mitral valve (38%) and the tricuspid valve (13%) [5]. Involvement of the prosthetic valve and multiple valves was reported in 13% of the cases [5]. Detectable vegetations were seen in 64% of cases on TTE [21]. Microbiologists should scrutinize positive blood cultures with Gram-positive pleomorphic cocci in pairs and short chains with slow growth or failure to grow for NVS [22]. Blood cultures subcultured within 48 hours yield a maximal growth on incubation in media [23]. For optimal growth, enriched medium with 0.001% pyridoxal or 0.01% L-cysteine is required [7]. Alternatively, growth of NVS satellite colonies can be improved with cross-streaking of the subculture plate with the helper bacteria *Staphylococcus aureus* [24]. The current recommendation is to use MALDI-TOF MS or any other automated system to identify NVS in the clinical microbiology lab for faster identification [25]. Automated systems are unable to determine the susceptibilities due to specific requirements. Broth microdilution minimum inhibitory concentration (MIC) testing in the cation-adjusted Mueller–Hinton broth with 2.5% to 5% lysed horse blood and 0.001% pyridoxal hydrochloride is the suggested method to complete antimicrobial susceptibility [7]. E test using Isosensitest agar supplemented with 5% defibrinated horse blood and 0.001% pyridoxal hydrochloride is an alternate rapid and more straightforward method when microdilution testing is not available [26].

Initial NVS endocarditis case series reported a higher mortality rate compared to enterococci or streptococcal viridans with a relapse rate of 17%, perivalvular abscess rate of 11%, mortality rate of 17%, and bacteriologic failure rate of 41% even after treatment with antibiotics that were effective in vitro, and 51% needed valve repair or replacement [5, 27, 28]. In vitro antibiotic sensitivity results are difficult to infer and clinically apply for an expected response to therapy. In vivo studies by Bouvet determined that vancomycin alone was significantly more effective than PCN and at least as effective as the combination of PCN and an aminoglycoside [29]. Although bactericidal activity of vancomycin was less than that of PCN, its concentration in the vegetations was higher than that of PCN, which explained the efficacy of the drugs in vivo. PCN with gentamicin and amikacin fell short of synergism, but the combination was more effective than PCN alone. In vivo results noted varied from those in vitro possibly due to different physiological states of NVS [29].

Literature review of NVS antimicrobial susceptibility [30–35] reveals that all studies are suggestive of an increased sensitivity of *G. adiacens* to PCN compared to *A. defectiva* but less susceptible to cephalosporins than *A. defectiva*. Amongst the ones wherein gentamicin sensitivity performed, three [31, 33, 35] of them showed no high-level resistance to aminoglycosides, whereas in one, high-level resistance to gentamicin was present [36]. In all studies, isolates were 100% susceptible to vancomycin. Quinolone susceptibility was more than 90% except in one study [36], wherein higher resistance to quinolones was seen with *G. adiacens*. 100% rifampin sensitivity was observed in three studies [31, 32, 35]. Carbapenem sensitivity was reviewed, and *G. adiacens* was more susceptible than *A. defectiva*. Three studies [34–36] showed some resistance to carbapenem seen in *A. defectiva* more than in *G. adiacens*. In 3 studies [33–35], susceptibilities for daptomycin and linezolid were reviewed, and daptomycin MIC was higher than noted for Gram-positive cocci, whereas NVS isolates were more susceptible to linezolid. 100% sensitivity was seen with tigecycline used in one study [34]. Current AHA (American Heart Association) guidelines recommend a combination of ampicillin or PCN plus gentamicin as for enterococcal infective endocarditis when the PCN MIC was ≥0.5 *µ*g/mL. A reasonable alternative is to use ceftriaxone combined with gentamicin. If vancomycin is used in patients intolerant of ampicillin or PCN, then the addition of gentamicin is not needed [36]. Recommendations in the ESC (European Society of Cardiology) 2015 Antibiotic Guideline include PCN G, ceftriaxone or, vancomycin for six weeks, combined with an aminoglycoside for at least first two weeks [37].

In our case, the patient had mitral valve vegetations with ruptured chordae tendineae and septic embolization to the brain leading to brain abscess and the L3-L4 spine resulting in discitis and osteomyelitis. IV meropenem was added after confusion and detection of brain abscess with improvement in the patient's clinical status. The patient has been successfully discharged after cardiac surgery on a six-week treatment course of IV vancomycin and ertapenem. Presumed *G. adiacens* prosthetic valve infective endocarditis without vegetations on TTE or emboli has been recently treated with eight weeks of IV vancomycin with success [38]. Treatment cure accomplished with vancomycin and gentamicin in a patient with septic embolization to the spleen and kidney [5]. Rifampin used along with vancomycin or PCN with gentamicin for infective endocarditis associated with prosthetic valve and pacemaker leads [5]. New antimicrobial susceptibility data displayed higher susceptibility rates for vancomycin, carbapenems, quinolones, and rifampin [32–35]. Since NVS isolates exhibit PCN tolerance, it cannot be substituted with cephalosporins for *G. adiacens* due to significant resistance, and a better replacement will be vancomycin [32–35]. Replacing with cephalosporins is an excellent choice if the NVS isolate is *A. defectiva* [32–35]. Also, gentamicin has been used successfully with vancomycin [5]. Aminoglycosides can be substituted with carbapenems or quinolones if side effects are a concern or with renal disease. PCN is not an ideal empirical agent due to significant resistance among all the NVS isolates [32–35]. Vancomycin with gentamicin or carbapenems or quinolones or rifampin will be an excellent empiric choice until NVS subspecies are detected and antimicrobial susceptibility obtained, as inappropriate empiric therapy can result in poor outcomes.

## 3. Conclusion

For NVS infective endocarditis, we suggest updates to treatment guidelines as there are significant antimicrobial susceptibility differences between the two most common agents *G. adiacens* and *A. defectiva*. Choosing an empirical agent or a combination is very important as delay leads to complications. We suggest not using PCN as an initial empiric agent. Based on clinical case reports and clinical experience attained in managing our patient, vancomycin monotherapy is an ideal empiric agent if the vegetations are small with no septic emboli. In more complicated case scenarios such as septic emboli, larger vegetations, valvular destruction, or metastatic abscesses, a better option would be a combination therapy of vancomycin with carbapenems or aminoglycosides or quinolones or rifampin. Excellent communication between the clinician and microbiologist is essential for early recognition. Blood cultures subcultured within 48 hours yield maximal growth. Treatment centers with limited resources, no automated systems, or the ability to isolate the organism should use referral facilities to obtain antimicrobial susceptibility results. For a successful cure, quick identification, timely initiation of empirical antibiotics, infectious disease consultation, and cardiac surgery when clinically indicated are necessary.

## References

[1] Liesman R. M., Pritt B. S., Maleszewski J. J., Patel R. (2017). Laboratory diagnosis of infective endocarditis. *Journal of Clinical Microbiology*.

[2] Roberts R. B., Krieger A. G., Schiller N. L., Gross K. C. (1979). Viridans *Streptococcal* endocarditis: the role of various species, including pyridoxal-dependent *Streptococci*. *Clinical Infectious Diseases*.

[3] Frenkel A., Hirsch W. (1961). Spontaneous development of *L* forms of *Streptococci* requiring secretions of other bacteria or sulphydryl compounds for normal growth. *Nature*.

[4] Collins M. D., Lawson P. A. (2000). The genus *Abiotrophia* (Kawamura et al.) is not monophyletic: proposal of *Granulicatella* gen. nov., *Granulicatella adiacens* comb. nov., *Granulicatella elegans* comb. nov. and *Granulicatella balaenopterae* comb. nov.. *International Journal Of Systematic and Evolutionary Microbiology*.

[5] Adam E. L., Siciliano R. F., Gualandro D. M. (2015). Case series of infective endocarditis caused by *Granulicatella* species. *International Journal of Infectious Diseases*.

[6] Cargill J. S., Scott K. S., Gascoyne-Binzi D., Sandoe J. A. T. (2012). *Granulicatella* infection: diagnosis and management. *Journal of Medical Microbiology*.

[7] Ruoff K. L. (1991). Nutritionally variant *Streptococci*. *Clinical Microbiology Reviews*.

[8] Kawamura Y., Hou X.-G., Sultana F., Liu S., Yamamoto H., Ezaki T. (1995). Transfer of *Streptococcus adjacens* and *Streptococcus defectivus* to *Abiotrophia* gen. nov. as *Abiotrophia adiacens* comb. nov. and *Abiotrophia defectiva* comb. nov., respectively. *International Journal of Systematic Bacteriology*.

[9] George R. H. (1974). The isolation of symbiotic *Streptococci*. *Journal of Medical Microbiology*.

[10] Bouvet A., Grimont F., Grimont P. A. D. (1989). *Streptococcus defectivus* sp. nov. and *Streptococcus adjacens* sp. nov., nutritionally variant *Streptococci* from human clinical specimens. *International Journal of Systematic Bacteriology*.

[11] Aas J. A., Paster B. J., Stokes L. N., Olsen I., Dewhirst F. E. (2005). Defining the normal bacterial flora of the oral cavity. *Journal of Clinical Microbiology*.

[12] Mikkelsen L., Theilade E., Poulsen K. (2000). *Abiotrophia* species in early dental plaque. *Oral Microbiology and Immunology*.

[13] Sato S., Kanamoto T., Inoue M. (1999). *Abiotrophia* elegans strains comprise 8% of the nutritionally variant *Streptococci* isolated from the human mouth. *Journal of Clinical Microbiology*.

[14] Siqueira J. F., Rôças I. N. (2006). *Catonella morbi* and *Granulicatella adiacens*: new species in endodontic infections. *Oral Surgery, Oral Medicine, Oral Pathology, Oral Radiology, and Endodontology*.

[15] Robertson D., Smith A. J. (2009). The microbiology of the acute dental abscess. *Journal of Medical Microbiology*.

[16] Yamaguchi T., Soutome S., Oho T. (2011). Identification and characterization of a fibronectin-binding protein from *Granulicatella adiacens*. *Molecular Oral Microbiology*.

[17] Okada Y., Kitada K., Takagaki M., Ito H. O., Inoue M. (2000). Endocardiac infectivity and binding to extracellular matrix proteins of oral *Abiotrophia* species. *FEMS Immunology and Medical Microbiology*.

[18] Senn L., Entenza J. M., Prod’hom G. (2006). Adherence of *Abiotrophia defectiva* and *Granulicatella* species to fibronectin: is there a link with endovascular infections?. *FEMS Immunology & Medical Microbiology*.

[19] Frehel C., Hellio R., Cremieux A.-C., Contrepois A., Bouvet A. (1988). Nutritionally variant *Streptococci* develop ultrastructural abnormalities during experimental endocarditis. *Microbial Pathogenesis*.

[20] Shailaja T. S., Sathiavathy K. A., Unni G. (2013). Infective endocarditis caused by *Granulicatella adiacens*. *Indian Heart Journal*.

[21] Steckelberg J. M., Murphy J. G., Ballard D. (1991). Emboli in infective endocarditis: the prognostic value of echocardiography. *Annals of Internal Medicine*.

[22] Padmaja K., Lakshmi V., Subramanian S., Neeraja M., Krishna S. R., Satish O. S. (2014). Infective endocarditis due to *Granulicatella adiacens*: a case report and review. *Journal of Infection in Developing Countries*.

[23] Gross K. C., Houghton M. P., Roberts R. B. (1981). Evaluation of blood culture media for isolation of pyridoxal-dependent *Streptococcus mitior* (mitis). *Journal of Clinical Microbiology*.

[24] Reimer L. G., Reller L. B. (1983). Effect of pyridoxal on growth of nutritionally variant *Streptococci* and other bacteria on sheep blood agar. *Diagnostic Microbiology and Infectious Disease*.

[25] Ratcliffe P., Fang H., Thidholm E., Boräng S., Westling K., Özenci V. (2013). Comparison of MALDI-TOF MS and VITEK 2 system for laboratory diagnosis of *Granulicatella* and *Abiotrophia* species causing invasive infections. *Diagnostic Microbiology and Infectious Disease*.

[26] Douglas C. P., Siarakas S., Gottlieb T. (1994). Evaluation of *E* test as a rapid method for determining MICs for nutritionally variant *Streptococci*. *Journal of Clinical Microbiology*.

[27] Brouqui P., Raoult D. (2001). Endocarditis due to rare and fastidious bacteria. *Clinical Microbiology Reviews*.

[28] Madison G., Golamari R., Bhattacharya P., Firstenberg M. S. (2018). Endocarditis caused by *Abiotrophia* and *Granulicatella* species. *Advanced Concepts in Endocarditis*.

[29] Bouvet A., Cremieux A. C., Contrepois A., Vallois J. M., Lamesch C., Carbon C. (1985). Comparison of penicillin and vancomycin, individually and in combination with gentamicin and amikacin, in the treatment of experimental endocarditis induced by nutritionally variant *Streptococci*. *Antimicrobial Agents and Chemotherapy*.

[30] Tuohy M. J., Procop G. W., Washington J. A. (2000). Antimicrobial susceptibility of *Abiotrophia adiacens* and *Abiotrophia defectiva*. *Diagnostic Microbiology and Infectious Disease*.

[31] Zheng X., Freeman A. F., Villafranca J. (2004). Antimicrobial susceptibilities of invasive pediatric *Abiotrophia* and *Granulicatella* isolates. *Journal of Clinical Microbiology*.

[32] Alberti M. O., Hindler J. A., Humphries R. M. (2015). Antimicrobial susceptibilities of *Abiotrophia defectiva*, *Granulicatella adiacens*, and *Granulicatella elegans*. *Antimicrobial Agents and Chemotherapy*.

[33] Mushtaq A., Greenwood-Quaintance K. E., Cole N. C. (2016). Differential antimicrobial susceptibilities of *Granulicatella adiacens* and *Abiotrophia defectiva*. *Antimicrobial Agents and Chemotherapy*.

[34] Prasidthrathsint K., Fisher M. A. (2017). Antimicrobial susceptibility patterns among a large, nationwide cohort of *Abiotrophia* and *Granulicatella* clinical isolates. *Journal of Clinical Microbiology*.

[35] Kanamoto T., Terakubo S., Nakashima H. (2018). Antimicrobial susceptibilities of oral isolates of *Abiotrophia* and *Granulicatella* according to the consensus guidelines for fastidious bacteria. *Medicines (Basel, Switzerland)*.

[36] Baddour Larry M., Wilson Walter R., Bayer Arnold S. (2015). Infective endocarditis in adults: diagnosis, antimicrobial therapy, and management of complications. *Circulation*.

[37] Habib G., Lancellotti P., Antunes M. J. (2015). 2015 ESC guidelines for the management of infective endocarditis. *European Heart Journal*.

[38] Jones B. M., Hersey R. M., Trestman I. J., Bland C. M. (2018). Successful treatment of a penicillin-intermediate and ceftriaxone-resistant *Granulicatella adiacens* presumed prosthetic valve endocarditis with vancomycin. *International Journal of Antimicrobial Agents*.

